# Supplementary material for: Dynamic modulation of inequality aversion in human interpersonal negotiations
Source: Commun Biol. 2022 Apr 14;5:359. doi: 10.1038/s42003-022-03318-8 (PMC9010408; doi:10.1038/s42003-022-03318-8)
Supplement: Supplementary file 2 — Supplementary Information [file 42003_2022_3318_MOESM2_ESM.pdf]

Supplementary Tables and Figures

Supplementary Table 1. **Demographic information of participants from the control experiment (n=25)**

Table 1.

Measure	Mean (SD)
Age	28.48 (10.19)
Gender	68% Female
Years of Education	17.68 (2.10)
QIDS-SR_{16}	4.96 (4.44)
State-STAI	27.56 (6.63)
Trait-STAI	34.96 (10.06)

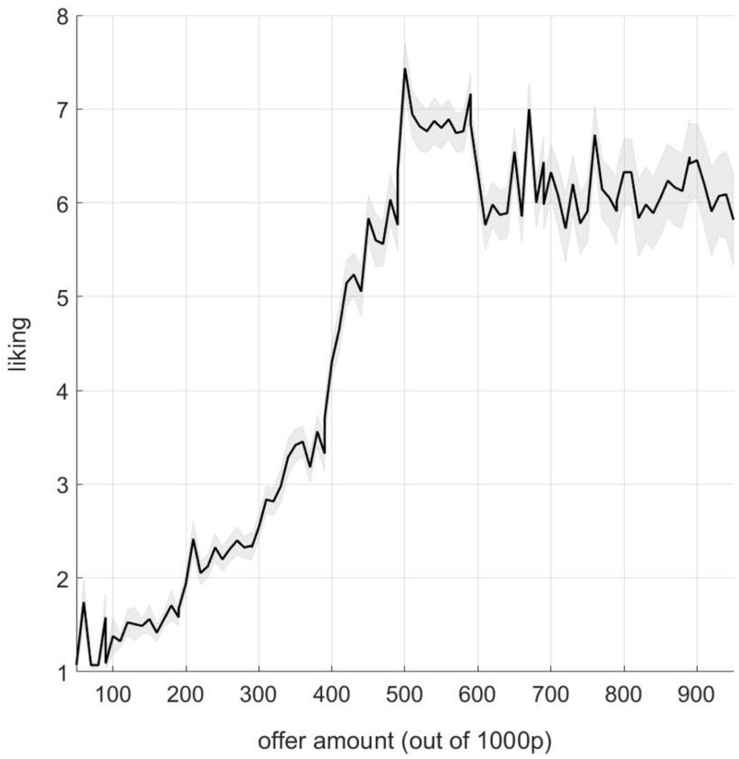

Supplementary Figure 1. Participants' liking ratings monotonically increase from 50p to 500p, which was the point of 50/50 split in the current experimental design. Relatively lower ratings for advantageous offers (>500p) indicate inequality aversion in this cohort. Wider SEM shading around the ratings of advantageous offers indicate a greater individual variability in terms of self-other inequality relative to how unfair offers were rated.

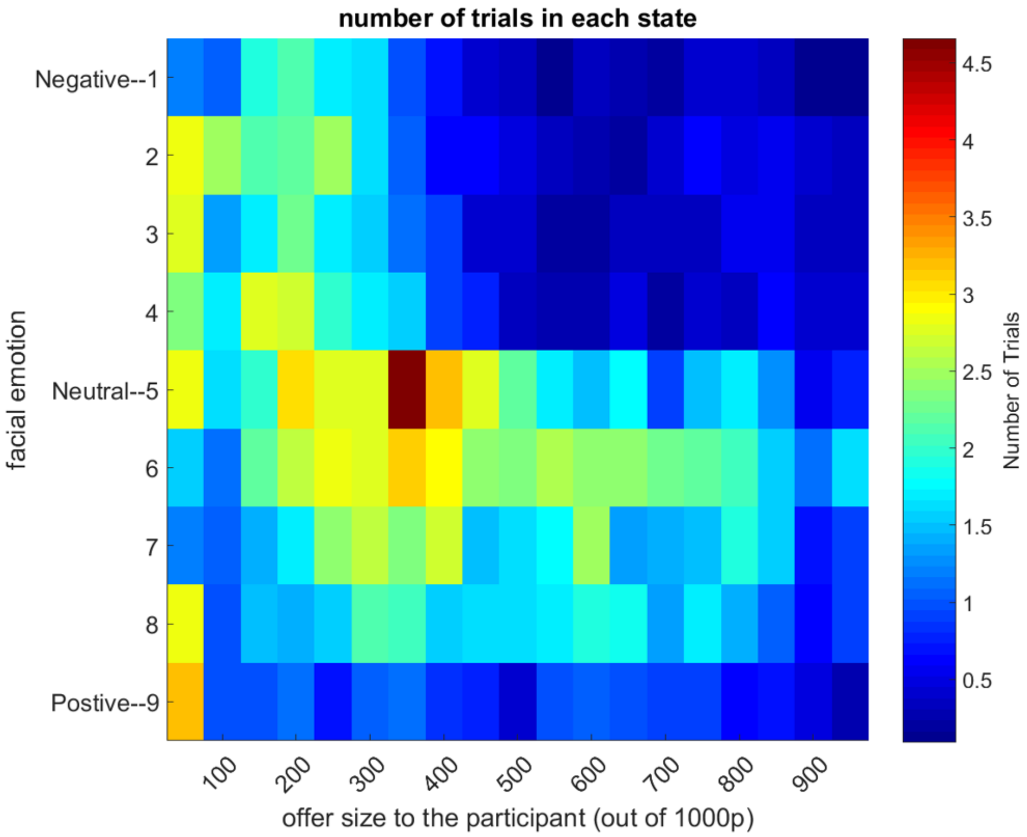

Supplementary Figure 2. Out of 240 trials, average number of trials participants spent in each state-space (i.e. combinations of proposer's facial emotion (y-axis) and offer amounts (x-axis)) represented as a heat map. Colour bar shows the number of trials. On average participants had 12.6±15.9 (mean±SD) trials in the upper right hand quarter (i.e. advantageous offers while the proposer is displaying a negative facial expression).

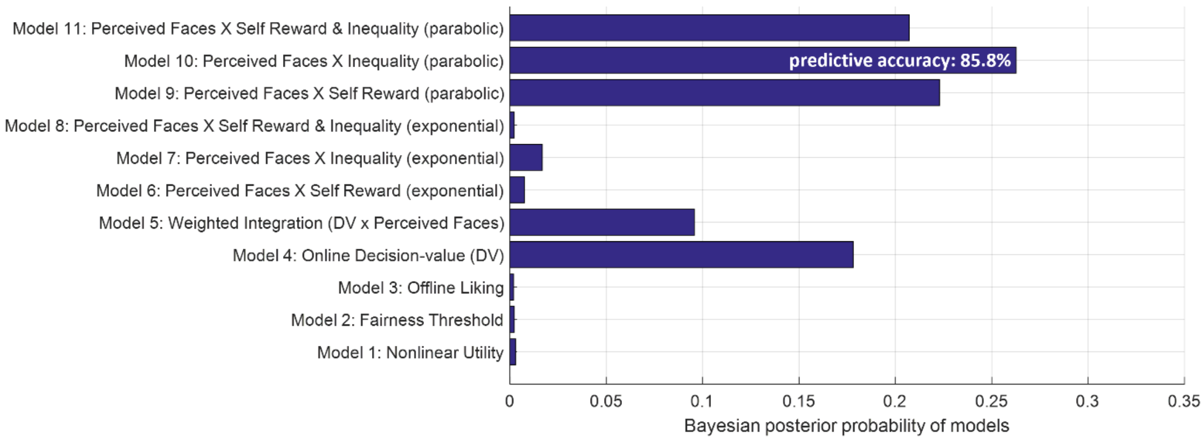

Supplementary Figure 3. Model selection implemented by feeding log-likelihood values from all models in a matrix format (number of participants x number of models) to spm_BMS.m (i.e. the Bayesian Model Selection script from SPM12 library), suggests that Model 10 is most likely to be the generative model for the observed data (based on model exceedance probabilities). This model suggests that perceived facial emotions selectively modulate the inequality term in a parabolic form.

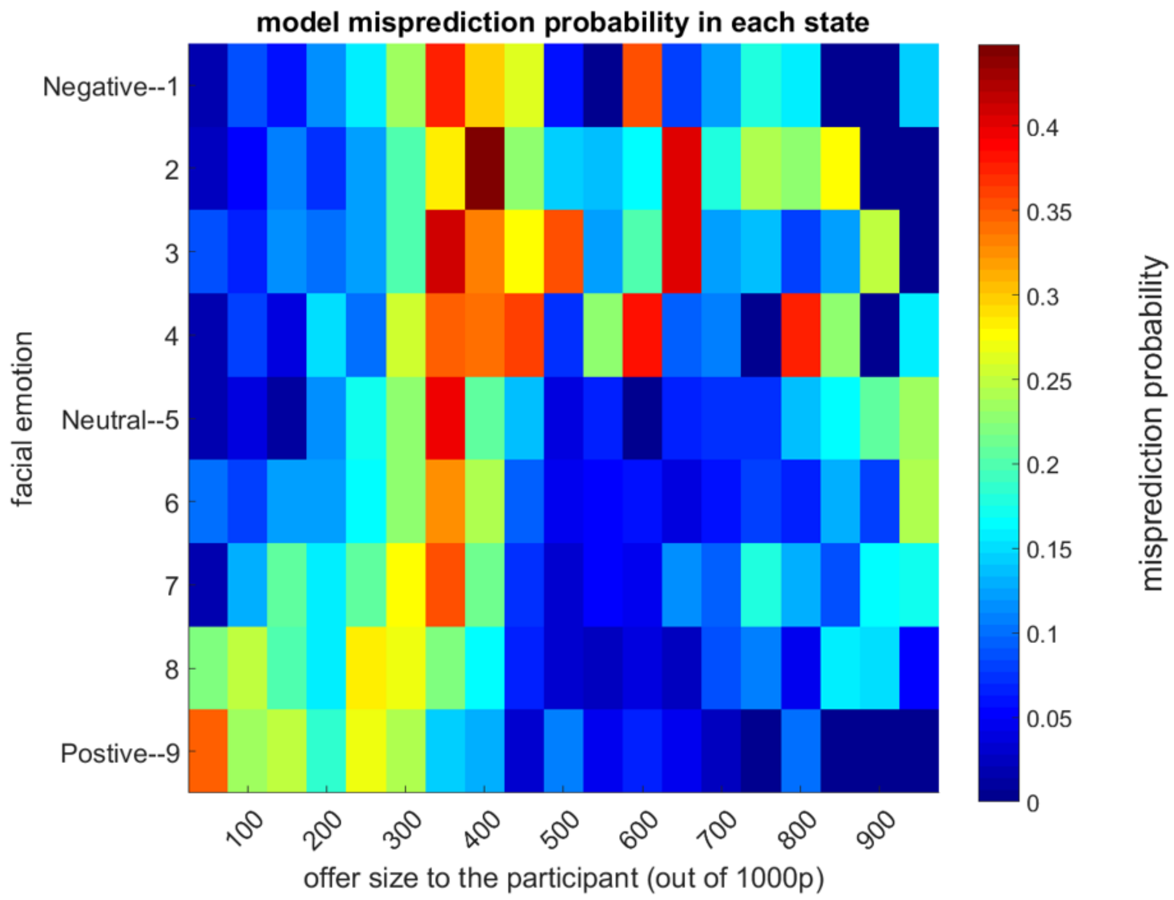

Supplementary Figure 4. Misprediction probabilities of the best-fitting model over the stimuli space. A regression analysis of the trials mispredicted by the best-fitting model did not show a significant main effect of proposer's facial emotions or the offer amount, indicating that the model does not miss the behavioural effect of faces and the offer amount (Figure 3) in a systematic manner.

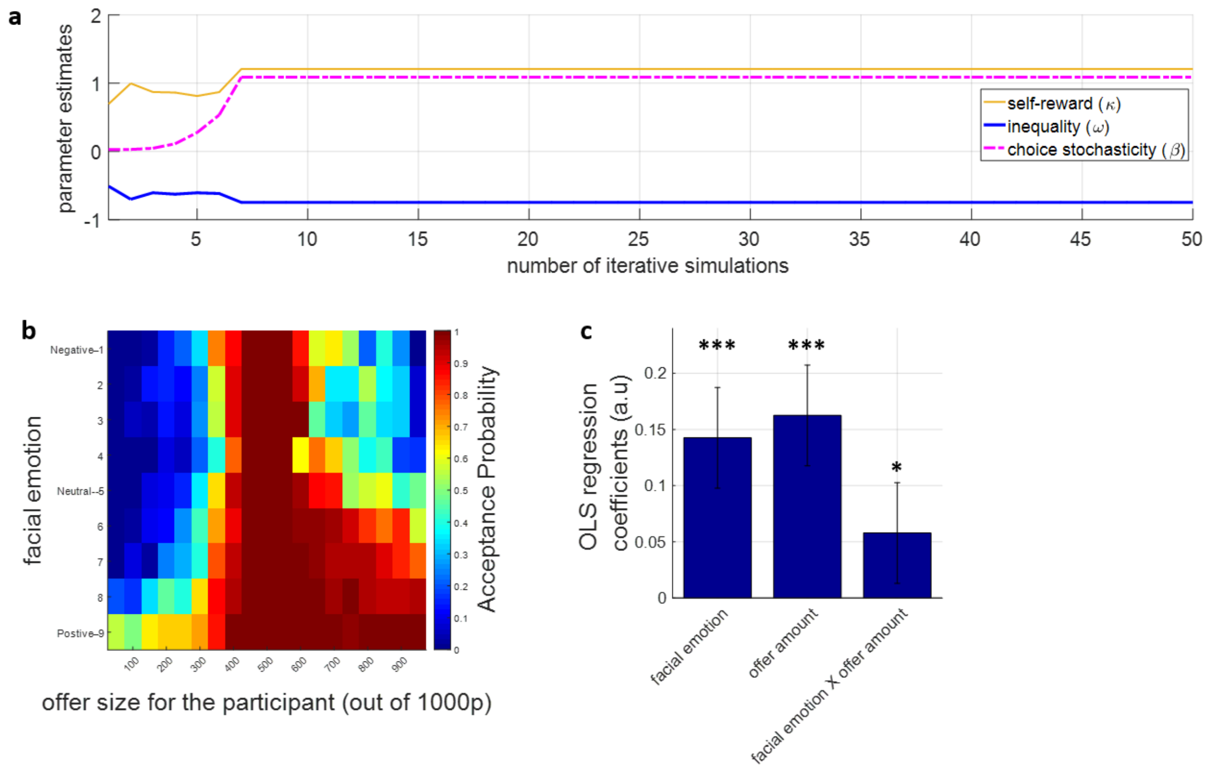

Supplementary Figure 5. Model recovery analyses. (a) A control analysis involving iterative simulations of the best-fitting model based on the population average of the model parameters on a task environment experienced by a representative subject demonstrate that parameter estimates quickly stabilise. In these simulations choices were generated stochastically by a sigmoid function and the parameters in the current simulation (i+1) were re-estimated based on the generated choices from the previous simulation (i). **(b-c)** Reanalysis of the raw data originally reported in Figure 3 by replacing participant choices with the choices generated by the best fitting dynamic inequality aversion model, is able to reproduce the essence of human behaviour and recapture significant main effects of facial emotions, offer amounts and their interaction (*P<0.05, ***P<.001).

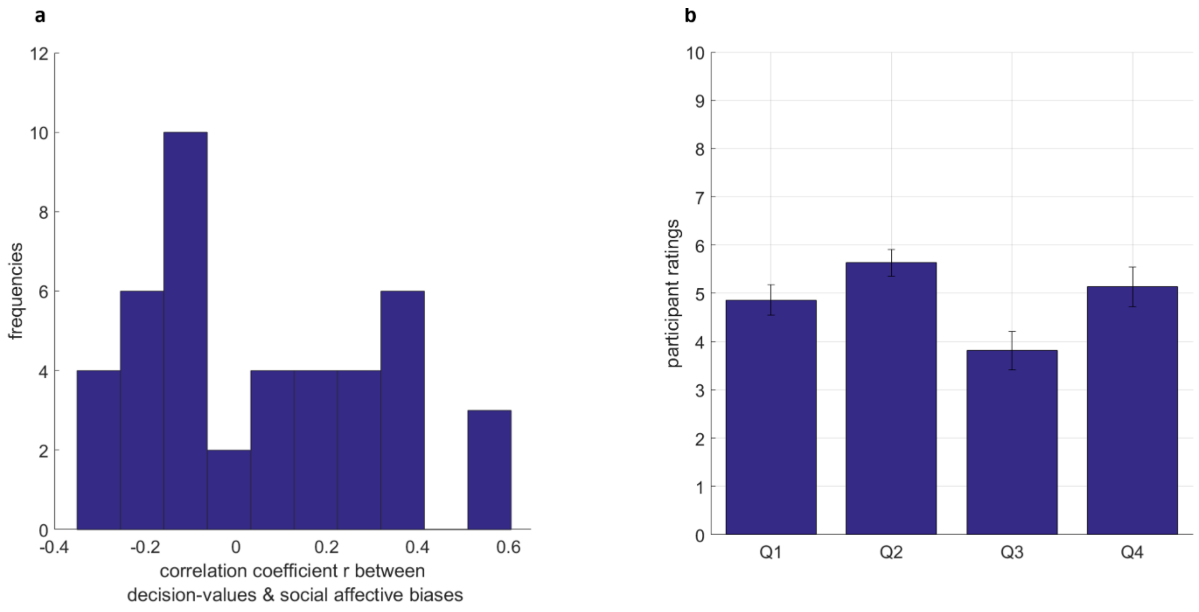

Supplementary Figure 6. Relationship between social affective biases and decision-values. (a) The distribution of correlation coefficients *r* between social affective biases and decision-values entered in to the pupillary model. Frequencies on the y-axis indicate the number of participants.

(b) Participants rated the proposer on 4 questions from 0 to 10.

Q1. How much do you think this person cares about rewards to others? (0: does not care at all, 10: cares very much)

Q2. How much would you like this person if you spent 1 hour with him/her in real-life? (0: strong dislike, 10: very likeable)

Q3. How many people do you know in real life who resembles this person (not physical appearance)? (number 0-10)

Q4. How socially close do you feel towards those people that you know? (0: not close at all, 10: very close).

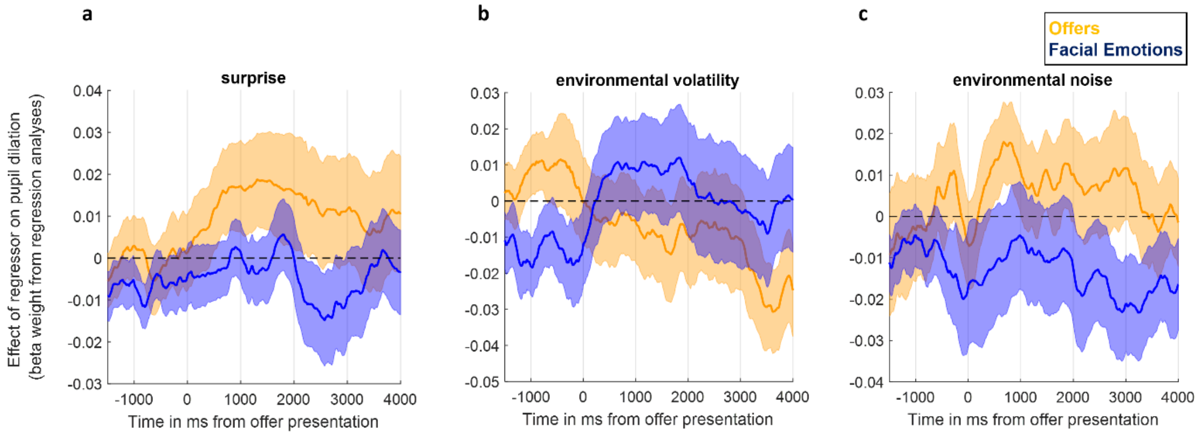

Supplementary Figure 7. Pupillary signal correlating with regressors estimated by the Bayesian filter. (a) Time evolution of coefficient estimates from the pupillary OLS regression model correlating with surprise associated with offer amounts and proposer's facial emotions. The surprise signal peaks around decision RT=1.74 seconds. **(b-c)** Time evolution of regression coefficients from the pupillary regression model for regressors describing environmental change. Pupil response was not statistically significant for any of these regressors. Error shading denotes ±1SEM.

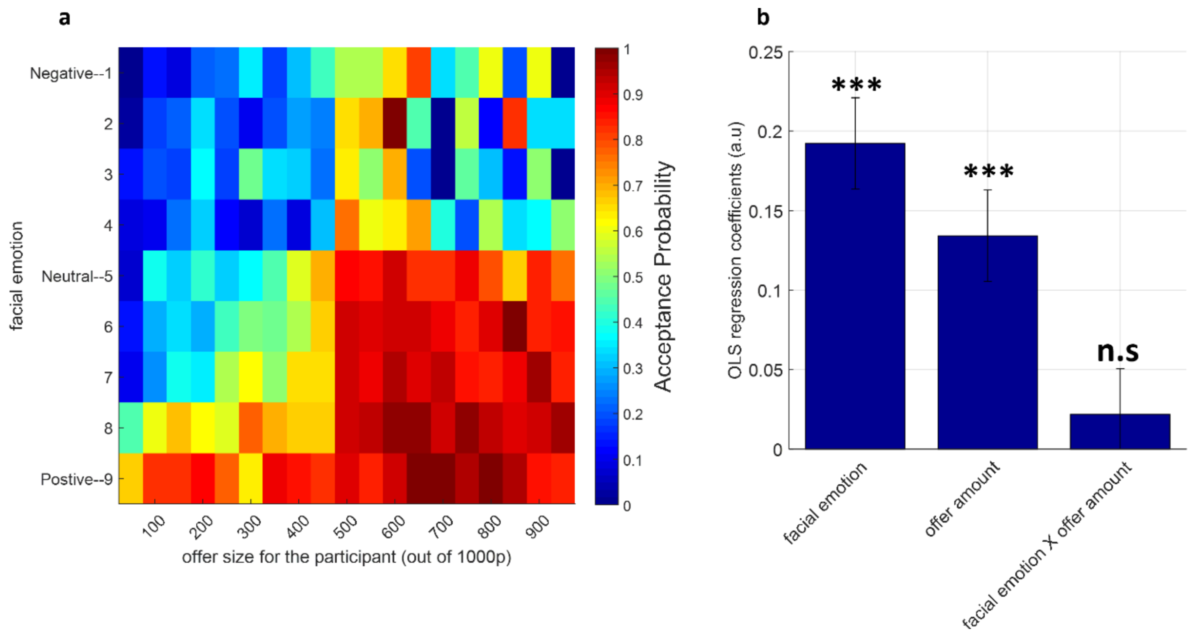

Supplementary Figure 8. Model-free results of the control experiment (n=25). (a) Participants' average acceptance probability across all combinations of proposer facial emotion (y-axis) and offer amount (x-axis) represented as a heatmap. Data shown is averaged across all participants (presented in the same manner as Figure 2 for the main experiment). Colour bar on the right shows the probability of accepting an offer. **(b)** A formal ordinary least squares (OLS) regression analysis conducted on acceptance probabilities indicated a significant main effect of proposer facial emotion ($t(24) = 13.225$, $p < 0.001$) and a significant main effect of offer amount ($t(24) = 9.231$, $p < 0.001$). There was no significant interaction between facial emotion and offer amount ($t(24) = 1.502$, $p = 0.135$). Note that error bars reflect ±1SEM. Logistic regression analysis of participant choice behaviour in the in-lab control experiment (akin to **Figure 4** for the main experiment) is reported in **Supplementary Figure 10**.

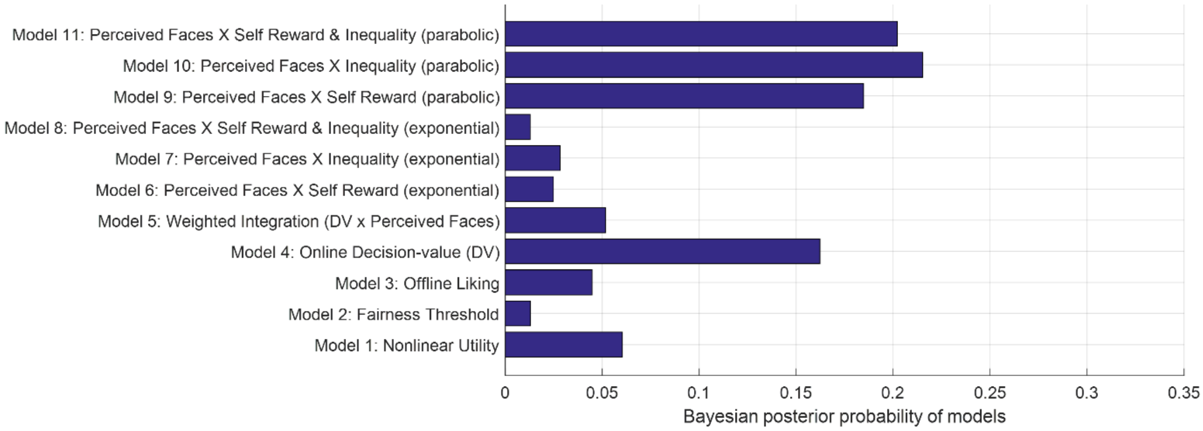

Supplementary Figure 9. As in the main experiment, model selection suggests that Model 10 is most likely to be the generative model for the observed data (based on model exceedance probabilities) in the iterative control experiment (n=25). This model suggests that perceived facial emotions selectively modulate the inequality term in a parabolic form. Globally parabolic family of models were better at explaining the data.

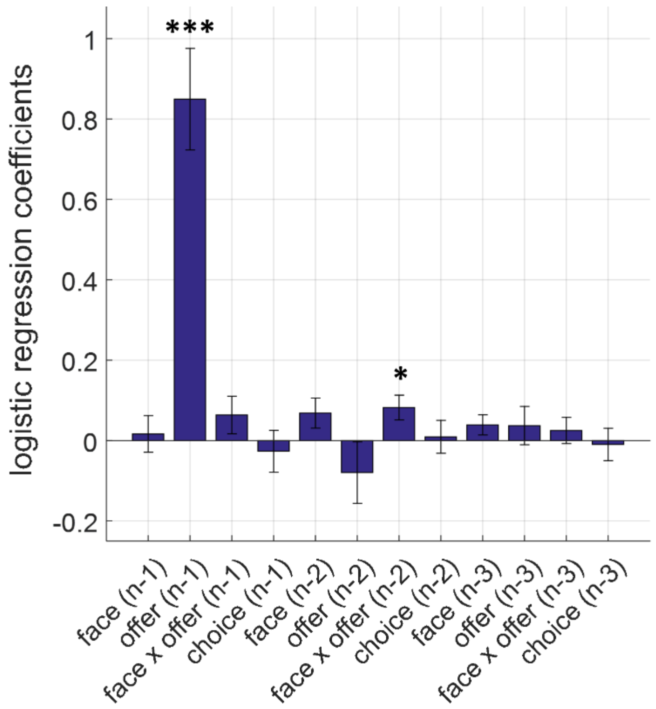

Supplementary Figure 10. Logistic regression analysis of participant choice behaviour in the control experiment (n=25). Coefficient estimates from the logistic regression model fitted to participant choices on the current (nth) trial indicates that offer amount from the n-1th trial significantly influence participant choice behaviour (***p<.001). This is the only regressor which had a similar contribution to participant choice on the nth trial with overlapping results from the main experiment (N=44). Error bars denote ±1 SEM. The average variance inflation factor computed for these 12 regressors was 1.0972.

Supplementary Materials.

Quantitative description of the computerised proposer strategy. Here we describe the transition probabilities of the computerised strategy which acted as the proposer. Similar probabilistic strategies have been used by other research groups to design opponent strategies in iterative social interactive decision-making games[1,2]. The description below illustrates the logic behind the computer strategy, the actual experimental materials are available from the authors upon request.

let *F* be the facial emotion of the proposer (range 1 (most negative) to 9 (most positive)).

let *O* be the offer amount (between 50 and 950p).

let *n* be the trial number (40 trials per block).

let *y* be the amount of offer change (either 50, 100 or 150p).

if n==1 **%first trial always start with a neutral face and a fair offer**

F=5; **% a neutral face**

O=500; **% a fair offer**

else **%after the first trial**

y=randsample([50 100 150],1,1,[1/3 1/3 1/3]);

%offer step either 50, 100 or 150 p with equal probability

end

if O <=500

if **reject count**➔	**0**	**1**	**2**	**3**	**4**	**5**	**>5**
**offer amount**							
**O-y**	.7	.6	.5	.25	.15	.1	0
**O**	.1	.15	.1	.1	.1	.1	.15
**O+y**	.2	.25	.4	.65	.75	.8	.85

elseif O>500

if **reject count**➔	**0**	**1**	**2**	**3**	**4**	**5**	**>5**
**offer amount**							
**O-y**	.7	.4	.6	.7	.75	.8	.85
**O**	.1	.2	.1	.1	.1	.1	.1
**O+y**	.2	.4	.3	.2	.15	.1	.05

end **%tables showing offer probabilities at each rejection level.**

```
if accept
        accept_count=accept_count+1;
        reject_count=0;
else
        accept_count=0;
        reject_count=reject_count+1;
end
```

if n>1 **%determines how faces are presented, cells showing probabilities**

if **reject count**➔	**0**	**1**	**2**	**3**	**4**	**5**	**>5**
**facial emotion**							
**F-1**	.1	.75					
**F**	.3	.15					
**F+1**	.6	.1					
**1**					.7	.7	
**2**				.7	.2	.3	.15
**3**			.5	.2	.1		.15
**4**			.35	.1			.35
**5**			.15				.35

if **accept count**➔	**1**	**2**	**3**	**4**	**5**	**>5**
**facial emotion**						
**5**	.25					.25
**6**	.55	.25				.45
**7**	.2	.55	.4	.15		.15
**8**		.2	.5	.35	.35	.15
**9**			.1	.5	.65	

end

```
% to prevent interaction being stuck in a limited range sample higher offer amounts if it is stuck
% in the fair offers range or if they are not sampled frequently enough
% (ie. at least 2 times within a block)
if sample_higher<2 && n>15
        if O>=400 && O<=500
                O=randsample ([O 750 800 850 900 950],1,1,[.1  .15 .25 .2 .15 .15]);
                % with 10% probability the offer stays the same
                sample_higher=sample_higher+1;
        end
end
if sample_higher<2 && n>30
        if O>=400 && O<=500
                O=randsample ([750 800 850 900 950],1,1,[.15  .25 .25 .2 .15]);
                sample_higher=sample_higher+1;
        end
end
```

Supplementary References:

1 McClure, E. B. *et al.* Responses to conflict and cooperation in adolescents with anxiety and mood disorders. *Journal of abnormal child psychology* **35**, 567-577 (2007).

2 Gradin, V. *et al.* Neural correlates of social exchanges during the Prisoner's Dilemma game in depression. *Psychological medicine* **46**, 1289-1300 (2016).

Example confederate pictures (higher numbers in .jpg files indicate positive valence):

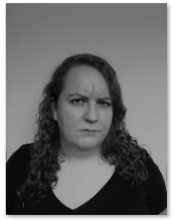
1.jpg

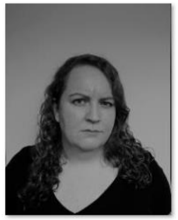
2.jpg

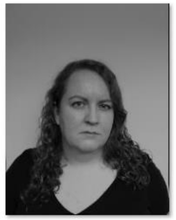
3.jpg

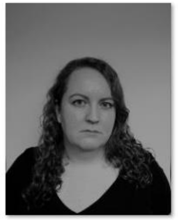
4.jpg

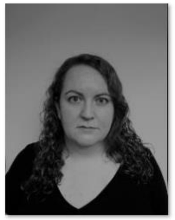
5.jpg

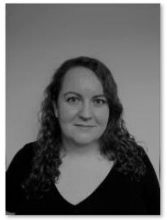
6.jpg

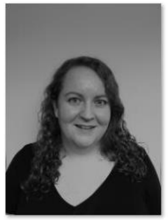
7.jpg

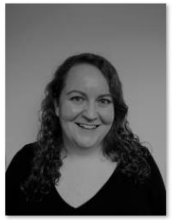
8.jpg

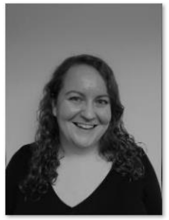
9.jpg

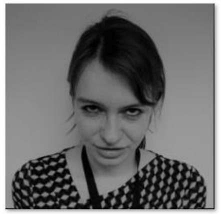
1.jpg

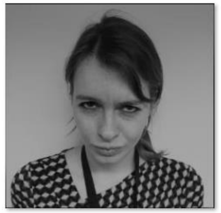
2.jpg

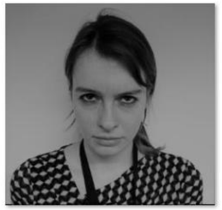
3.jpg

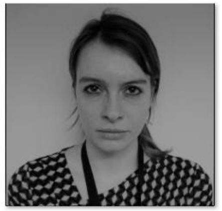
4.jpg

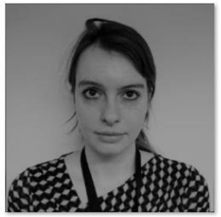
5.jpg

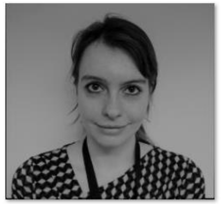
6.jpg

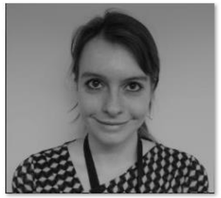
7.jpg

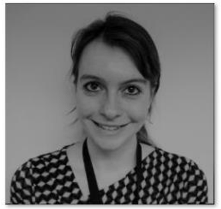
8.jpg

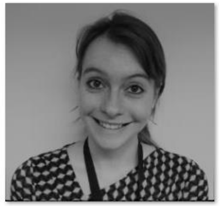
9.jpg
