# Supplementary material for: Dynamic modulation of inequality aversion in human interpersonal negotiations
Source: Commun Biol. 2022 Apr 14;5:359. doi: 10.1038/s42003-022-03318-8 (PMC9010408; doi:10.1038/s42003-022-03318-8)
Supplement: Supplementary file 3 — Description of Additional Supplementary Files [file 42003_2022_3318_MOESM3_ESM.pdf]

Description of Additional Supplementary Files

File name: Supplementary Data 1
Description: Source data underlying the graphs in the manuscript.
